# Integrated Metabolomics and Proteomics Analyses in the Local Milieu of Islet Allografts in Rejection versus Tolerance

**DOI:** 10.3390/ijms22168754

**Published:** 2021-08-15

**Authors:** Luis F. Hernandez, Luis R. Betancourt, Ernesto S. Nakayasu, Charles Ansong, Gerardo A. Ceballos, Daniel Paredes, Midhat H. Abdulreda

**Affiliations:** 1 Diabetes Research Institute, University of Miami Miller School of Medicine, Miami, FL 33136, USA; 2 Knoebel Institute for Healthy Aging, University of Denver, Denver, CO 80208, USA; betahitcher@gmail.com (L.R.B.); gerardoacv@gmail.com (G.A.C.); Daniel.Paredes@du.edu (D.P.); 3 Department of Morphological Sciences, Faculty of Medicine, School of Medicine, University of Los Andes, Mérida 5101, Venezuela; 4 Biological Sciences Division, Pacific Northwest National Laboratory, Richland, WA 99352, USA; ernesto.nakayasu@pnnl.gov (E.S.N.); cansong07@gmail.com (C.A.); 5 Center of Biomedical Engineering and Telemedicine, Faculty of Engineering, University de Los Andes, Mérida 5101, Venezuela; 6 Department of Electrical and Computer Engineering, DU, Denver, CO 80208, USA; 7 Department of Neurology, Johns Hopkins University, Baltimore, MD 21287, USA; 8 Department of Surgery, University of Miami Miller School of Medicine, Miami, FL 33136, USA; 9 Department of Microbiology and Immunology, University of Miami Miller School of Medicine, Miami, FL 33136, USA; 10 Department of Ophthalmology, University of Miami Miller School of Medicine, Miami, FL 33136, USA

**Keywords:** anterior chamber of the eye, allogeneic islet transplant, intraocular transplant, immune regulation, LC-MS (liquid chromatography-mass spectrometry), MEKC-LIFD (micellar electrokinetic chromatography with laser induced fluorescence detection), metabolomics, M1 macrophages, M2 macrophages, pancreatic islets, proteomics, Type 1 diabetes, T1D, Tregs (T regulatory cells), Teff (T effector cells), rejection, tolerance, Warburg effect

## Abstract

An understanding of the immune mechanisms that lead to rejection versus tolerance of allogeneic pancreatic islet grafts is of paramount importance, as it facilitates the development of innovative methods to improve the transplant outcome. Here, we used our established intraocular islet transplant model to gain novel insight into changes in the local metabolome and proteome within the islet allograft’s immediate microenvironment in association with immune-mediated rejection or tolerance. We performed integrated metabolomics and proteomics analyses in aqueous humor samples representative of the graft’s microenvironment under each transplant outcome. The results showed that several free amino acids, small primary amines, and soluble proteins related to the Warburg effect were upregulated or downregulated in association with either outcome. In general, the observed shifts in the local metabolite and protein profiles in association with rejection were consistent with established pro-inflammatory metabolic pathways and those observed in association with tolerance were immune regulatory. Taken together, the current findings further support the potential of metabolic reprogramming of immune cells towards immune regulation through targeted pharmacological and dietary interventions against specific metabolic pathways that promote the Warburg effect to prevent the rejection of transplanted islets and promote their immune tolerance.

## 1. Introduction

Cancer cells and immune cells (e.g., macrophages and lymphocytes) switch their metabolism under inflammatory conditions towards aerobic glycolysis and diminish oxidative phosphorylation while obtaining their ATP for survival and maintenance. This metabolic switching was first described in 1926 by Otto Von Warburg and has become known as the “Warburg effect” [1,2,3,4]. In immune cells, the Warburg effect occurs in response to cues in their microenvironment (e.g., cytokines and metabolites) and has a direct influence on their functional polarization towards inflammation versus immune regulation/suppression. This highlights the potential for immune cell regulation by manipulating their metabolism (i.e., immunometabolism), a process referred to as metabolic reprogramming [5]. It is now established that immune cell survival, activation, and functional polarization also depend on specific metabolic pathways of, not only carbohydrates (glucose), but also fatty acids, amino acids and their by-products (e.g., polyamines), proteins as well as other cofactors such as vitamins, minerals, and metals (e.g., iron). For example, interfering with polyamine production from arginine by difluoromethylornithine (DFMO) diminishes T effector (Teff) cell proliferation and pro-inflammatory polarization by Th1/Th17 cytokines [6]. Macrophages reduce their aerobic glycolysis and increase oxidative phosphorylation, fatty acid oxidation, and mitochondrial biogenesis upon anti-inflammatory signaling by Th2 cytokines such as IL-4 (interleukin-4) [7].

Up- and down-regulation of different proteins determines the pathway(s) that will predominate during the immune metabolic response [8,9,10,11,12]. Together, these observations underscore the importance of fully understanding the metabolism of immune cells and the opportunities such understanding offers to manipulate immune responses in the context of various immune conditions. This is also critical in therapeutic transplant applications where the success of such therapies depends on immune reactions that dictate the transplant outcome either by promoting the survival and longevity of the graft or its rejection by the host immune system.

Substantial evidence shows that the functional polarization of innate and adaptive immune cells by Th1/Th17 cytokines (T-helper type 1/17) towards pro-inflammatory phenotypes, such as M1 macrophages and Teff lymphocytes, is key in the rejection of allogeneic (allo) grafts [13,14,15,16]. Alternatively, their Th2 (T-helper type 2) polarization towards regulatory phenotypes such as M2 macrophages and T regulatory (Treg) cells promotes allograft survival and immune tolerance. Thus, manipulating immune cell polarization via immunometabolism, or metabolic reprogramming, is promising in transplant therapies to ensure the graft survival and efficacy on the long-term [17,18,19]. To this effect, our approach in this and prior works has involved two complementary innovations: (1) establishing the immune privileged anterior chamber of the eye (ACE) as a new site for islet transplant [20,21,22,23] and (2) gaining direct access to the islet graft’s immediate microenvironment—represented in the aqueous humor (AQH)—to understand the local metabolic and protein profiles in the context of the immune outcome, i.e., graft rejection versus tolerance [24,25].

Capitalizing on these technical capabilities of the ACE-platform, we previously identified significant changes in the local proteome and metabolome in association with intraocular islet allografts rejection [25,26]. Here, we further examine specific changes in local metabolites and proteins involved in immunometabolism in the context of immune tolerance. We performed integrated metabolomics and proteomics analyses in the AQH of mice that either rejected or tolerated their intraocular islet allografts. The new findings revealed significant changes in the local levels of several free amino acids, small primary amines, and soluble proteins that are all related to the Warburg effect and with direct links to immune cell function and polarization.

## 2. Results

### 2.1. Patterns of Electropherograms Generated in Aqueous Humor Samples during Rejection versus Tolerance Are Significantly Different

**Figure 1:**
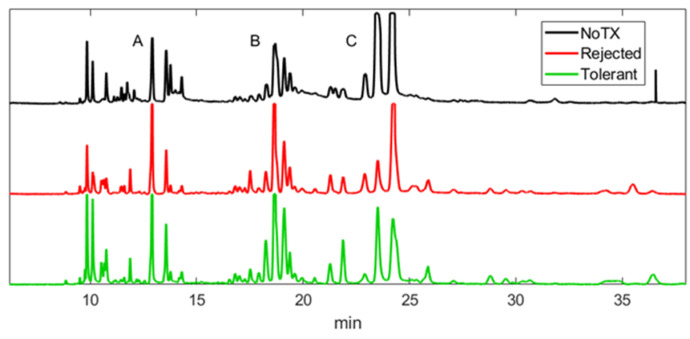
Representative electropherograms (EPGs; shown as voltage versus time in minutes) generated in aqueous humor (AQH) samples obtained from non-transplanted control mice (NoTX; black) and transplant recipients that either rejected (red) or tolerated (green) their intraocular islet allografts. The EPGs showed distinct peak patterns across the experimental groups. Three groups of peaks (corresponding to amino acids and small primary amines) and some ghost peaks were consistently distinguished in each EPG and were designated as group A, B and C. Although the experimental conditions of animals (i.e., NoTX, rejected, tolerant) had a similar general pattern, the number of individual features/metabolites (peaks) and their abundance (peak amplitude/area) within each group were very different among the experimental groups.

Electropherograms (EPGs) generated by MEKC-LIFD (micellar electrokinetic chromatography with laser induced fluorescence detection) analysis in AQH samples obtained from mice that either rejected or tolerated their intraocular islet allografts and from non-transplanted controls showed different patterns in three distinct groups of peaks (groups A, B, and C) based on their migration time (Figure 1). The migration of each peak, which correspond to a putative metabolite, is dictated by the electro-osmotic properties of the metabolite [27,28]. Therefore, peaks in different EPGs with the same migration time likely correspond to the same metabolite [26]. The first group of peaks (group A) migrated between 6 and 15 min. The metabolites in this group (amino acids and small primary amines) are likely cations and hydrophilic molecules with low octane/water partition coefficient such as lysine, which is positively charged at the running buffer pH of 8.7 and has a 0.00003 partition coefficient. The second group (group B) migrated between 15 and 20 min and the metabolites in this group should be neutral and slightly lipophilic, such as phenylalanine with neutral charge and a partition coefficient of 0.023. The last group (group C) migrated beyond the 20 min time point; this group typically consists of highly lipophilic and anionic molecules such as aspartic acid with negative charge and a partition coefficient of 0.375. The different patterns in each peak group in the EPGs derived from non-transplanted controls and transplant recipients that either rejected or tolerated their intraocular islet allografts are suggestive of significant changes in the local metabolome and proteome under these different experimental conditions. 

### 2.2. Free Amino Acids Levels in the Local Microenvironment of Rejected versus Tolerated Islet Allografts

**Figure 2:**
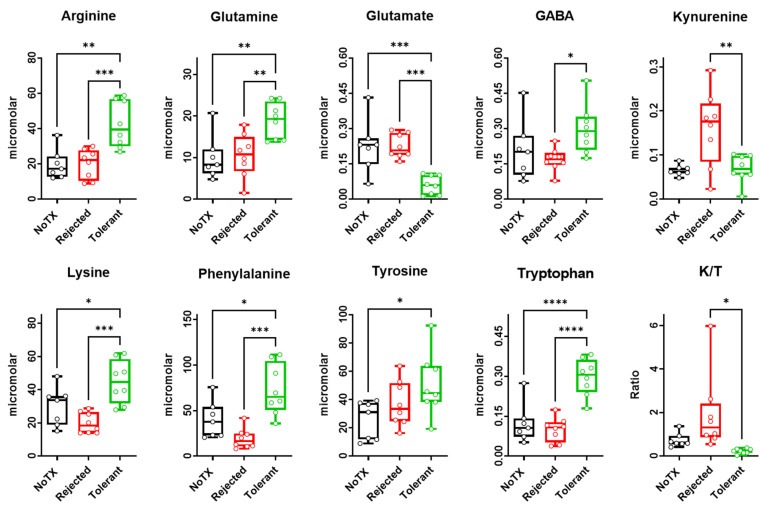
Several metabolites were identified in the electropherograms (EPGs) of aqueous humor (AQH) samples and had significantly different concentrations in the of non-transplanted controls (NoTX; black) and the transplant recipients that either rejected (red) or tolerant (green) their intraocular islet allografts. Concentrations were measured as indicated above and in Methods section based on the spiked standard solutions, and data were shown as box and whisker plots indicating the medians (horizontal lines in each box) and the upper and lower quartiles with all data points shown as open round symbols corresponding to each sample from one mouse (*n* = 7 mice for NoTX and *n* = 8 mice for rejected and tolerant). Asterisks denote significant difference by ANOVA followed by Tukey’s multiple comparison test with * *p* < 0.05; ** *p* < 0.001; *** *p* < 0.0001; **** *p* < 0.00001 (also see Table 1 for more details).

We performed targeted metabolomics analysis by MEKC-LIFD in the immediate microenvironment (represented by AQH) of pancreatic islets transplanted in the ACE of allogeneic recipient mice that either rejected or tolerated their grafts, as well as non-transplanted controls (NoTX) as a baseline reference. We identified and measured the concentrations of free amino acids and small primary amines by iteratively spiking the samples with standard solutions of a known metabolite and concentration. We identified this way eight amino acids and kynurenine (a metabolite of the tryptophan metabolism pathway). The EPGs were processed without alignment to measure the concentration of targeted metabolites in the sample groups [26]. The peak of the known analyte in the standard solution corresponded to a concentration of 6 micromolar; thus, we calculated the concentration of each targeted metabolite by dividing its peak area in the experimental samples by the area of the corresponding peak in the standard solution. The identified eight amino acids were arginine, glutamine, glutamate, gamma aminobutyric acid (GABA), lysine, phenylalanine, tyrosine, and tryptophan (Figure 2). We also calculated the kynurenine to tryptophan (K/T) ratio by dividing the kynurenine concentration by that of tryptophan. Each metabolite was compared in the three experimental groups (i.e., NoTx, rejected, and tolerant) by ANOVA followed by pairwise comparisons using Tuckey’s multiple comparison test. All nine metabolites had statistically significant differences across the experimental groups (see Table 1).

### 2.3. Warburg Effect-Related Protein Levels in the Local Microenvironment of Pancreatic Islet Allografts Are Significantly Different in Rejection versus Tolerance 

**Figure 3:**
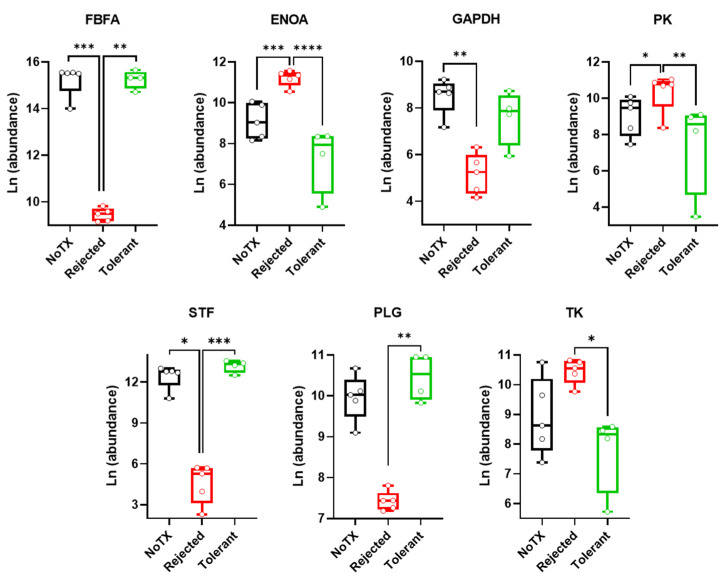
Relative abundance of proteins related to the Warburg effect measured in aqueous humor (AQH) samples of non-transplanted controls (NoTX; black) and transplant recipient mice that either rejected (red) or tolerated (green) their intraocular islet allografts. Relative abundance values were log-transformed using the natural log (Ln), and data were shown as box and whisker plots indicating the medians (horizontal lines in each box) and the upper and lower quartiles with all data points shown as open round symbols corresponding to each sample from one mouse (*n* = 5 mice for NoTX; *n* = 5 mice for rejected; and *n* = 4 mice for tolerant). Asterisks denote significant difference by ANOVA followed by Tukey’s multiple comparison test with * *p* < 0.05; ** *p* < 0.001; *** *p* < 0.0001; **** *p* < 0.00001 (see Table 2).

We performed untargeted proteomics analysis by LC-MS/MS (liquid chromatography-mass spectrometry) in AQH samples obtained from transplant recipient mice that either rejected or tolerated their intraocular islet allografts as well as from non-transplanted controls for comparison. Several proteins directly or indirectly related to the Warburg effect and inflammation were identified and quantified among 170 proteins measured in all samples without exception (see Table S1 in Supplementary Materials for a complete list of identified proteins). The proteins related to the Warburg effect included fructose bisphosphate aldolase (FBFA), alpha enolase (ENOA), glyceraldehyde-3-phosphate dehydrogenase (GAPDH), pyruvate kinase (PK), serotransferrin (STF), plasminogen (PLG; plasmin heavy chain A), and transketolase (TK). Figure 3 shows side-by-side comparisons of each protein across the non-transplanted control group (NoTX) and the transplant recipients that either rejected or tolerated their allografts. All shown proteins had some significant difference in their relative abundance in the local microenvironment of the islet allografts (represented in AQH) across the experimental groups as indicated. Comparisons were done by ANOVA followed by Tukey’s multiple comparison test (also see Table 2). The data showed that STF, PLG, FBFA, and GAPDH significantly decreased and, conversely, ENOA and PK increased in the rejected group when compared with the NoTX controls. None were significantly different in the tolerant group when compared with controls. However, all seven proteins changed in opposite directions in the tolerant and rejected groups with six out of seven showing significant differences in rejection versus tolerance.

## 3. Discussion

We recently reported on an artificial intelligence (AI) and machine learning (ML) approach to predict the immune outcome (i.e., rejection or tolerance) of pancreatic islet transplants in allogeneic recipient mice solely based on pattern recognition of unidentified peaks in EPGs generated by MEKC-LIFD [26]. We showed that several putative extracellular metabolites in the immediate microenvironment of intraocular islet allografts (represented in the AQH) were significantly different in tolerant recipient mice when compared to rejecting ones and non-transplanted controls. We also proposed that such metabolites could provide candidate biomarkers of islet graft rejection versus tolerance. Here, we performed further targeted metabolomics analyses to determine the identity of such metabolites not only as potential biomarkers but, more importantly, to (a) gain conceptual insight into the metabolic mechanisms associated with either transplant outcome and (b) explore new possibilities for immune cell metabolic reprogramming to prevent rejection of islet transplants and promote their immune tolerance. We also performed untargeted proteomics in AQH samples under the same conditions and integrated the analyses to investigate the simultaneous changes in the local proteome and metabolome in association with rejection versus tolerance. The results showed significant changes in the local levels of several amino acids, small primary amines, and proteins related to the Warburg effect with direct links to immune cell polarization and function depending on the transplant outcome. Notably, the mice in this study were fed normal diet without supplementation of any kind; therefore, the observed changes in these metabolites and proteins were strictly the consequence of either the immune rejection or tolerance of the pancreatic islet allografts.

Consistent with prior evidence showing arginine to have anti-inflammatory effects by decreasing IL-1β, increasing superoxide dismutase (SOD) and decreasing the death receptor FAS expression [29,30]; and phenylalanine inhibiting antibody production and promoting tolerance of skin grafts [31,32], we found here that arginine and phenylalanine concentrations were significantly higher in the AQH of the tolerant recipients when compared to those that rejected their intraocular islet grafts and to non-transplanted controls (Figure 1 and Table 1). We also found that lysine concentration was significantly lower in association with islet rejection, which is consistent with clinical data in serum of patients who rejected their kidney transplants [33] and preclinical data showing increased pro-inflammatory cytokines and reduced regulatory ones upon feeding of low lysine diet in piglets [34]. The latter report also showed that lower lysine levels can lead to elevated plasma IgG/IgM (immunoglobulin G/M) and upregulation of nuclear factor kappa-light-chain-enhancer of activated B cells (NF-kB), toll-like receptors (TLRs), and extracellular signal-regulated protein kinases (ERK)1/2 signaling and, consequently, increased inflammation in several critical metabolically active organs. Alternatively, higher lysine levels were shown to enhance IL-10 production and Treg proliferation in cultures of feline T lymphoblasts [35]. In a mouse model of pancreatitis, oral administration of lysine also decreased the plasma concentration of IL-6, malonaldehyde, and nitric oxide, and increased the key antioxidant enzymes superoxide dismutase, catalase, and glutathione peroxidase in the pancreas [36].

Moreover, in a model of cerebral inflammation induced by the administration of hemine to primary cultures of microglia, lysine increased the levels of arginase-1 (Arg-1), CD206 (cluster of differentiation 206), and Ym1 which are markers of immune regulatory M2 macrophages [37]. Consistently, intracerebral injection of hemine caused a strong inflammatory reaction and neuronal apoptosis; however, administration of hemine together with lysine reduced apoptosis in a dose-dependent fashion. In addition, lysine promotes the expression of microRNA-557 which suppresses the expression of PTEN (phosphate and tension homology deleted on chromosome ten), a transcription factor that enhances the polarization of microglia towards the pro-inflammatory M1 phenotype. Interestingly, PTEN systemic knockout in transgenic *pten^flox-flox^* mice reduced inflammation in adipose tissues and insulin resistance caused by high-fat-diet (HFD) [38]. Taken together, this line of evidence clearly shows that lysine plays an essential role in reducing inflammation and promoting the functional polarization of immune cells towards immune regulation. Thus, the augmented concentration of lysine we observed in the AQH of recipient mice that tolerated their intraocular islet grafts likely promoted tolerance. These combined findings highlight the potential of lysine administration in islet transplant to prevent immune rejection and promote tolerance.

Consistently with arginine, phenylalanine and lysine, tryptophan was also markedly increased in the AQH of the tolerant mice compared with rejecting ones, and there was no difference between the latter and the non-transplanted controls. Since we did not supplement the mouse diet in these studies with any amino acid, the increase in the tryptophan levels could be attributed to a blockade in the metabolic pathways that utilize it. Tryptophan can potentially be (1) utilized as a building block of proteins; (2) metabolized through the tryptophan–kynurenine pathway; and (3) converted to serotonin, melatonin, and some other monoamines. The tryptophan–kynurenine pathway has been investigated in the context of organ transplant and the kynurenine/tryptophan (K/T) ratio increases in inflammation and transplant rejection [39]. Consequently, a decrease in the K/T ratio is considered anti-inflammatory, consistent with immune regulation and tolerance [40]. Here, we found that the K/T ratio in the AQH of mice with intraocular islet grafts was significantly higher in the rejecting recipients (by ANOVA) and lower (*p* < 0.05 by *t*-test) in tolerant ones when compared to non-transplanted controls; thus, demonstrating that the tryptophan–kynurenine pathway is actively upregulated in rejection and downregulated during tolerance. 

In addition, the increased levels of extracellular tryptophan in the tolerant mice makes it more available for the third metabolic pathway, i.e., its conversion into bioactive amines such as serotonin, which has trophic effects on islets [41,42]. Indeed, the limiting step in serotonin synthesis depends on the availability of tryptophan [43]. Therefore, the observed increase in tryptophan is consistent with a pro-survival local milieu in association with the graft tolerance. Together, these findings further highlight the potential for metabolic reprogramming of the immune system through tryptophan supplementation together with the administration of inhibitors of enzymes that convert tryptophan to kynurenine (i.e., tryptophan 2,3 dioxygenase [TDO] and indoleamine 2,3 dioxygenase [IDO]) to promote immune tolerance of transplanted allogeneic islets.

Our studies also showed that glutamine was significantly increased in the tolerant recipients when compared to rejecting ones and non-transplanted controls. Glutamine plays a prominent role in immune responses because it controls the proliferation of macrophages and T cells and the production of IL-2 at the early stages of inflammation [44,45]. It also has anti-inflammatory effects [46,47]. Therefore, its elevated levels in the local milieu of the tolerated islet allografts are consistent with an immune regulatory role promoting the graft tolerance. One possible mechanism contributing to this increase in glutamine levels in the extracellular space is through the downregulation of the glutaminolytic pathway (i.e., glutamine conversion to glutamate) in the absence of the Warburg effect and in association with the polarization of immune cells towards immune regulation during the induction and maintenance of the islet allograft tolerance [7,48,49]. Notably, reduced consumption of a metabolic substrate would lead to an increase in its levels and associated reduction in its by-products. Indeed, in addition to the increased glutamine levels, we found that glutamate was significantly reduced in the AQH of the tolerant mice (Figure 2).

It should be noted, however, that glutamate is also synthesized by islet alpha cells where it is typically released together with glucagon [50,51] and has autocrine and paracrine effects on the pancreatic islet function [52,53,54]. Therefore, it is likely that the glutamate we measured in the AQH is contributed to by the intraocular islet grafts as well as nerve fibers within the ACE and other eye structures (e.g., retina) [55]. However, the fact that glutamate levels were the same in rejecting recipients and non-transplanted controls suggests that the reduction in glutamate levels in association with the islet graft tolerance is primarily related to metabolic reprogramming of the immune cells that led to reduced utilization of its substrate (glutamine) and, consequently, its own production. Furthermore, it should be noted that high levels of glutamate are deleterious to cells by two mechanisms: (1) excitotoxicity due to increased free intracellular calcium and (2) increased free radicals, both of which induce apoptosis [56]. Therefore, the lower glutamate levels in the local milieu of the tolerated islet allografts is consistent with an environment favorable to their survival and maintenance.

Moreover, we found a significant increase of GABA concentration in the AQH of the tolerant mice when compared to the rejected group, and there was no difference between them and the non-transplanted controls, although, a trend for increased GABA was evident. GABA is a neurotransmitter with known effects on the immune system and inflammation [57,58]. GABA and the enzymes essential for its production and breakdown (i.e., glutamic acid decarboxylase [GAD] and GABA transaminase [GABA-T]) are present in the beta cells of pancreatic islets [59,60,61,62], and the presence of plasma anti-GAD autoantibodies is a hallmark of T1D [63,64]. Thus, reduced GABA consequent to anti-GAD autoimmunity is implicated in T1D pathogenesis [65]. Normally, the main sources of GABA in the AQH are the plasma [66] and some amacrine and ganglionic neurons of the retina [67,68]. In our model, the intraocular islet grafts also likely contribute to extracellular GABA in the AQH [69] and this may have contributed to the higher GABA levels in the tolerant animals with intact islets. Therefore, we cannot at this time determine whether the significantly elevated GABA levels in the AQH in association with tolerance contributed to immune regulation and tolerance of the intraocular islet allografts. On the other hand, although anti-GAD autoantibodies are likely not present in our current model of allogeneic islet transplant, such autoantibodies may play a role in non-transplanted T1D patients by diminishing GAD activity and local GABA production in the eye; which could explain in part the inflammatory damage to the retina in diabetes. 

Furthermore, in addition to its potential involvement in immune regulation, GABA plays an important role in autocrine and paracrine signaling within pancreatic islets [70,71]. GABA inhibits glucagon release from alpha cells [72] and stimulates insulin secretion via autocrine signaling through the GABA-A receptor in the beta cells [69,73]. It has also been shown that GABA has protective effects on beta cells in a T1D murine model and in human islets under inflammatory conditions [74]. Therefore, the increased GABA in the tolerant mice likely contributed, at the least, to the homeostasis of the tolerated islet allografts. It has also been suggested that GABA promotes the proliferation of beta cells [75,76], which could explain the increased size of tolerated islet grafts (not shown) that we reported on previously [77,78]. Therefore, the use of GABA as a co-adjuvant factor in newly diagnosed T1D patients to promote beta cell mass recovery and in islet transplant applications to promote the survival and function of transplanted islets might be warranted for further investigation [79].

As noted earlier, we performed untargeted proteomics analyses in AQH samples from different groups of mice (i.e., rejected, tolerant, and NoTX controls) (Figure 3) and we integrated the local changes in the proteome and metabolome in the context of the Warburg effect and inflammation in association with rejection versus tolerance. The results showed that fructose-bisphosphate aldolase A (FBFA) significantly decreased in the rejected group. FBFA has been shown to reduce the Warburg effect and inflammation and prevent T1D in the NOD (non-obese diabetic) mouse model [80]. Therefore, it is likely that the decrease of FBFA promoted inflammation and increased the vulnerability of the transplanted islets during rejection. By contrast, alpha enolase (ENOA) significantly increased in the rejected group. ENOA catalyzes the conversion of 2-phosphoglycerate (2PG) to phosphoenolpyruvate (PEP) and, therefore, it is upregulated during the Warburg effect. It also promotes the polarization of macrophages to the pro-inflammatory M1 phenotype [11] and is upregulated on the surface of macrophages to promote inflammation [81]. Therefore, the increase of ENOA in the local microenvironment of the islet grafts likely contributed to their rejection.

Moreover, glyceraldehyde-3-phosphate dehydrogenase (GAPDH) catalyzes the conversion of glyceraldehyde 3-phosphate (G3P) to 1,3-biphosphoglycerate (1,3BPG) during glycolysis. GAPDH is considered anti-inflammatory [82] and, therefore, its decrease can be associated with inflammation. Here, we found that GAPDH decreased in mice that rejected their intraocular islet allografts. Notably, GAPDH is upregulated by glutamine [83], and we found that glutamine was significantly upregulated in tolerant mice compared to the rejected and control counterparts. This suggests that glutamine might have also contributed to immune tolerance in our model by enhancing the availability of GAPDH. Interestingly, it has been shown that protein caspase-1 inhibits GAPDH gene transcription [84]. This inhibition of the glycolytic pathway deprives cells of energy sources and contributes to their death. There is also evidence suggesting that the inhibition of GAPDH by the compound GB111-NH2 activates the inflammasome in macrophages to induce pyroptotic cell death [85]. Therefore, this evidence together with our current findings suggest another mechanism by which low levels of GAPDH might have contributed to the rejection of islet allografts in our model.

Another enzyme in the glycolytic pathway is pyruvate kinase (PK). Our results showed that PK was significantly increased in the rejected group compared to tolerant recipients and NoTX controls. PK catalyzes the conversion of PEP into pyruvate (Pyr) producing ATP in the process. Overexpression of PK is one of the mechanisms of inducing the Warburg effect [86,87,88]. Glycolytically-shifted LPS-activated M1 macrophages consistently show upregulation of the PK isoform PKM2. However, PKM2 is inactive as a dimer and only as a tetramer can it catalyze the PEP-Pyr conversion. PKM2 dimers also directly bind HIF-1α (hypoxia-inducible factor 1-alpha) to stabilize it and induce the transcription of pro-inflammatory cytokines (e.g., IL-1b) and promote macrophage M1 polarization [9]. Alternatively, lower PK levels attenuate the Warburg effect and induce IL-10 production and promote the polarization of M2 macrophages with anti-inflammatory, immune regulatory function [89,90,91]. Therefore, our results showing significantly reduced local PK levels are consistent with immune regulation in the graft microenvironment in association with the graft tolerance in our studies.

Our data also showed significantly reduced serotransferrin (STF; aka transferrin) in rejected recipients compared to tolerant and NoTX counterparts (Figure 3). STF is a glycoprotein that transports iron in the plasma [92]. In general, STF is constitutively expressed and cells needing iron express the transferrin receptor and internalize the transferrin-bound iron by receptor-mediated endocytosis [93]. Therefore, a decrease in STF is suggestive of its active uptake by cells [94]. Macrophages express the transferrin receptor by which they contribute to recycling iron from senescent red blood cells. Activated M1 macrophages kill bacteria by depriving them of iron in their microenvironment [95]. Iron is also essential for mammalian cell function and survival [96]. Moreover, high uptake of iron by macrophages from the local milieu might enhance their expression of iron-dependent enzymes such as IDO [97], which catalyzes the tryptophan-kynurenine metabolism pathway in association with graft rejection [98]. Indeed, our data showed significantly elevated K/T ratio in the AQH of rejected recipients. The opposite was also observed in tolerance with an evident trend towards lower K/T ratios and higher STF levels in the tolerant recipients (*p* < 0.05 by *t*-test). Therefore, the significantly lower STF levels in the local milieu of the rejected islet grafts is suggestive of active involvement of pro-inflammatory macrophages through another mechanism involving iron deprivation of the islet graft cells.

Furthermore, the current studies revealed another enzyme, transketolase (TK), which was significantly higher in the AQH of mice who rejected their intraocular islet allografts when compared to tolerant recipients. TK promotes the Warburg effect as it accelerates the conversion of glucose to lactate via the pentose phosphate pathway [99]. Conversely, plasminogen (PLG), which diminishes the Warburg effect and promotes immune regulatory M2 macrophages [100] was significantly higher in tolerant recipients.

Taken together, the current experimental findings indicated that several metabolic pathways converged to promote the Warburg effect during immune-mediated rejection of islet allografts; and that several of such pathways were downregulated in association with the graft tolerance, in addition to new ones that promoted immune regulation and the survival and function of the transplanted pancreatic islets.

## 4. Materials and Methods

### 4.1. Animals

All animal experiments were conducted in accordance with the guidelines and approval of the Institutional Animal Care and Use Committee (IACUC) of the University of Miami (protocol # 17-171; approved 25 September 2017). Mice were purchased from the Jackson Laboratory (Bar Harbor, ME, USA) and housed in cages with free access to food and water within facilities under the supervision of the Department of Veterinary Resources (DVR). After reaching the experimental endpoint, the animals were euthanized, and aqueous humor (AQH) and tissue samples were collected for later analysis. Euthanasia was performed either by carbon dioxide or anesthesia (by isoflurane) overdose followed by cervical dislocation, as recommended by the American Veterinary Medical Association (AVMA) for the species.

### 4.2. Islet Isolation and Transplantation

Pancreatic islets were isolated by enzymatic digestion of donor pancreata of male DBA/2 mice, followed by density gradient separation/purification using protocols standardized at the Diabetes Research Institute (DRI) Pre-Clinical Cell Processing and Translational Models Core [101]. Isolated islets were cultured overnight and then implanted in the anterior chamber of the eye (ACE) of C57BL/6 (B6) recipient mice under full anaesthesia by isoflurane as previously described in detail [20]. Each mouse received 10–20 IEQ (islet equivalents) in one eye. The transplanted mice were divided into two groups, one group received a transient immune intervention with anti-CD154/CD40L (cluster of differentiation 154/40 ligand) monoclonal antibody (clone MR-1) peri-transplant to induce immune tolerance of the allogeneic islet grafts, and the other group received isotype Ig control, as previously described in detail [77].

### 4.3. Monitoring the Survival and Rejection of the ACE-Transplanted Islets

We used a full MHC-mismatch model of islet transplantation (DBA/2 donor islets into B6 recipients). As previously described in detail [102], survival of the ACE-transplanted islets in the recipient mice was assessed by quantitative volumetric measurements of the individual islets based on their backscatter (reflection) of a 633 nm laser. In brief, engrafted islets were mapped in digital images acquired during the first week after transplant and the same islets were revisited during the longitudinal studies post-transplant. Structural integrity of the islets was documented by direct visualization and in high-resolution digital images, and their survival was assessed by quantitative volumetric analysis in confocal micrographs acquired in the backscatter/reflection mode. Three-dimensional (3D) confocal micrographs of individual ACE-transplanted islets in each mouse were acquired using 20× magnification, 0.5 numerical aperture (NA) water immersion objective in z-stacks spanning the full height of each islet. The quantitative volumetric analysis of the individual islets was performed in 3D using Volocity software (Quorum Technologies; Puslinch, ON, Canada; more information about the software can be found at http://quorumtechnologies.com/volocity), as we previously described [25,102]. The analysis was performed in rejected and tolerant recipient mice. When transplanted islets in a mouse lasted more than 100 days post-transplant (>post-operative day [POD]100) with their average volume remaining ≥70% relative to their respective baselines, the graft in that mouse was considered tolerated [77]. A mouse was considered rejected when the average volume of its islets decreased <70% before POD100 [102]. Typically, mice rejected their intraocular islet grafts within 3–4 weeks post-transplant and only those treated with MR-1 tolerated their allografts [77]. AQH samples were collected at the time when a recipient mouse was deemed to have rejected or tolerated its islet allograft as defined above.

### 4.4. Aqueous Humor (AQH) Samples

As we previously described in detail [24,25], samples of AQH were collected from all mice under isoflurane anesthesia by penetrating the anterior chamber of the eye laterally through the cornea with the tip (~40 µm in diameter) of a pulled glass capillary tube (1.17 inside diameter and 1.5 mm outside diameter). Between 4 and 6 µL of AQH were extracted by capillarity action and were frozen at −80 °C for later analysis by MEKC-LIFD or LC-MS. Samples from rejected recipients were collected at the time of rejection onset (±3 days). Samples from tolerant recipients were collected after confirming tolerance (i.e., >POD100).

### 4.5. Sample Preparation and Metabolomics Analysis by MEKC-LIFD 

The AQH samples were derivatized for micellar electrokinetic chromatography with laser induced fluorescence detection (MEKC-LIFD) analysis as reported elsewhere in detail [26,27]. In brief, a 2.5 mM fluorescein isothiocyanate isomer I (FITC) in acetone solution was mixed 1:1 (*v*/*v*) with 20 mM carbonate buffer at pH 10.0. Two microliters of this derivatizing solution were mixed with 2 microliters of AQH. After 24 h in the dark the mixtures were ready for MEKC-LIFD analysis.

Targeted metabolomics analysis by MEKC-LIFD was performed using a homemade instrument described elsewhere in detail [27,28]. A 60 cm long fused silica was filled with a sodium tetraborate (40 mM) and sodium dodecylsulphate (20 mM) buffer at pH 8.7. The silica capillary had a 25 and 350 µM inside and outside diameter, respectively. The samples/derivatizing solution mixtures were hydrodynamically loaded at the anodic end of the capillary by applying a −12 PSI pressure (vacuum) at the opposite cathodic end of the capillary. An electric field was created by applying +27 KV at the anodic end of the capillary while keeping the cathodic end grounded. The bands were detected by a collinear detector equipped with a 488 nm continuous wave laser focused through a 64×, 0.85 NA objective on a 1 cm window made at 40 cm of the anodic end of the capillary by burning off the polyimide cover. The fluorescence was collected with the same objective through a long-pass filter centered at 505 nm and focused with a 10× eyepiece on the window of a photomultiplier tube (PMT) to measure fluorescence intensity that was converted into voltage in the electropherograms (EPG). The signal was collected at 40 points per second using a homemade software and data output in the form of voltage versus time was presented as EPGs. Unknown analytes were identified by iterative spiking of the samples/derivatizing solution mixtures. This consisted of separately derivatizing a 1 mg/mL solution of one known analyte at a time by adding five microliters of derivatizing solution to obtain a standard solution. Then, the samples/derivatizing solution mixtures were with this standard solution at 1:1 (*v*/*v*) ratio and run by MEKC-LIFD as described above. Consequently, all the peaks decreased to half of their amplitude, but the peak of the spiked unknown analyte increased and, hence, was identified and its concentration calculated as described below. Notably, the concentration of the unknown analyte in the standard solution was in the low millimolar (mM) range (~5 mM) while the concentration of the FITC was 6 micromolar (µM) in the 1:1 derivatized sample/standard solution mixture. Since FITC concentration is ~3 orders of magnitude less than that of the analyte to identify, FITC became the limiting reactant and, thus, the peak amplitude/area of the now identified analyte in the standard solution corresponded to 6 µM of the fluorescein isothiocarbamate derivative. This allowed calculating the concentration of the unknown analyte in the derivatized sample according to the following equation:

$$\left[Analyte\right]=\frac{area\;of\;identified\;peak\;in\;sample}{area\;of\;peak\;in\;standard}\times \;6$$

where [*Analyte*] is the concentration of the identified analyte in micromolar (µM).

### 4.6. Sample Preparation and Proteomics Analysis by LC-MS/MS

AQH samples were processed and proteomics analysis by LC-MS/MS were conducted as previously described in detail [25]. The LC-MS/MS data were analyzed with MaxQuant software (v1.6.5.0, Max Planck Institute of Biochemistry) as detailed elsewhere [103]. In brief, peptides were identified by searching against the mouse reference proteome databased from UniProt Knowledge Base (downloaded 14 August 2018 from https://www.uniprot.org/). The parameters of the search included cleaving by trypsin in both peptide termini but allowing up to two undigested sites per peptide. The cysteine carbamidomethylation was set as a fixed modification and the protein *N*-terminal acetylation and methionine oxidation were considered as variable modifications. The error tolerance in the mass was set as the default parameter of the software. Extraction of the quantitative information was done using the method of intensity-based absolute quantification (iBAQ) as described previously [104].

## 5. Conclusions

In summary, the findings in the current report shed new light on several immune metabolic mechanisms that are distinctly active within the local microenvironment of pancreatic islet allografts during their rejection versus tolerance. Several metabolic pathways respectively in inflammation or immune regulation were involved. Specific free amino acids, small primary amines, and soluble proteins key in the Warburg effect were identified and had consistent expression patterns in association with the transplant outcome. These included, but were not limited to, the tryptophan–kynurenine and arginine–polyamines pathways, the glutamine–glutamate and GABA synthesis pathways, the pro-inflammatory cytokine-inducing pyruvate kinase pathway, the iron uptake/metabolism pathways, and the GAPDH–inflammasome and ENOA–macrophage M1-polarizing pathways. The results also indicated that the observed changes in the local metabolome and proteome are likely mediated primarily by infiltrating immune cells, which raises the possibility for immune cell manipulation through metabolic reprogramming by targeted pharmacological and/or dietary interventions to promote the immune tolerance of pancreatic islet allografts.

## Figures and Tables

**Table 1:** Tuckey’s multiple comparisons for metabolites (amino acids and small primary amines) identified in the immediate local microenvironment of islet allografts under the various experimental conditions.

Metabolite	F(2/22)	R Squared	*p*-Value	Tol vs. Rej	Tol vs. NoTX	Rej vs. NoTX
Arginine	12.32	0.552	0.0003	***	**	*ns*
Glutamine	7.784	0.4377	0.0032	**	**	*ns*
Glutamate	13.55	0.5755	0.0002	***	***	*ns*
GABA	3.744	0.2724	0.0416	*	*ns*	*ns*
Kynurenine	7.685	0.4354	0.0033	**	*ns*	**
Lysine	11	0.5238	0.0006	***	*	*ns*
Phenylalanine	13.33	0.5713	0.0002	***	*	*ns*
Tyrosine	3.685	0.2693	0.0434	*ns*	*	*ns*
Tryptophan	22.78	0.6949	<0.0001	****	****	*ns*
K/T ratio	5.336	0.3479	0.0139	*	*ns*	*ns*

* *p* < 0.05; ** *p* < 0.001; *** *p* < 0.0001; **** *p* < 0.00001; *ns*: non-significant with *p* > 0.05.

**Table 2:** Tuckey’s multiple comparisons for proteins related to the Warburg effect identified in the immediate local microenvironment of islet allografts under the various experimental conditions.

Protein	F(2/11)	R Squared	*p*-Value	Tol vs. Rej	Tol vs. NoTX	Rej vs. NoTX
Fructose-bisphosphate aldolase A (FBFA)	16.28	0.7475	0.0005	**	*ns*	***
Alphaenolase (ENOA)	29.7	0.8438	<0.0001	***	*ns*	****
Glyceraldehyde-3-phosphate dehydrogenase (GAPDH)	7.962	0.5915	0.0073	*ns*	*ns*	**
Pyruvate kinase (PK)	8.484	0.6067	0.0059	**	*ns*	*
Serotransferrin (STF)	17.3	0.7588	0.0004	***	*ns*	*
Plasminogen (PLG)	9.401	0.6309	0.0042	**	*ns*	*ns*
Transketolase (TK)	6.951	0.5583	0.0112	*	*ns*	*ns*

* *p* < 0.05; ** *p* < 0.001; *** *p* < 0.0001; **** *p* < 0.00001; *ns*: non-significant with *p* > 0.05.

## Data Availability

The proteomics LC-MS/MS data are provided in the supplementary information of this article. The MEKC-LIFD data are freely available upon request.

## Supplementary Materials

The following are available online at https://www.mdpi.com/article/10.3390/ijms22168754/s1.
